# Health need and the use of alternative medicine among adults who do not use conventional medicine

**DOI:** 10.1186/1472-6963-10-220

**Published:** 2010-07-29

**Authors:** Richard L Nahin, James M Dahlhamer, Barbara J Stussman

**Affiliations:** 1 National Center for Complementary and Alternative Medicine, National Institutes of Health, 6707 Democracy Blvd., Bethesda, Maryland, 20892-5475, USA; 2 National Center for Health Statistics, Centers for Disease Control and Prevention, 3311 Toledo Road, Hyattsville, Maryland, 20782, USA

## Abstract

**Background:**

We hypothesize that a substantial portion of individuals who forgo conventional care in a given year turn to some form of alternative medicine. This study also examines whether individuals who use only alternative medicine will differ substantially in health and sociodemographic status from individuals using neither alternative medicine nor conventional care in a given year. To identify those factors that predict alternative medicine use in those not using conventional care, we employed the socio-behavioral model of healthcare utilization.

**Methods:**

The current study is a cross-sectional regression analysis using data from the 2002 National Health Interview Survey. Data were collected in-person from 31,044 adults throughout the 50 states and the District of Columbia.

**Results:**

19.3% of adults (38.3 million) did not use conventional care in a 12 month period, although 39.5% of these individuals (14.7 million) reported having one or more problems with their health. Of those not using conventional care, 24.8% (9.5 million) used alternative medicine. Users of alternative medicine had more health needs and were more likely to delay conventional care because of both cost and non-cost factors compared to those not using alternative medicine. While individual predisposing factors (gender, education) were positively associated with alternative medicine use, enabling factors (poverty status, insurance coverage) were not.

**Conclusions:**

We found that a quarter of individuals who forgo conventional care in a given year turn towards alternative medicine. Our study suggests that the potential determinants of using only alternative medicine are multifactorial. Future research is needed to examine the decision process behind an individual's choice to use alternative medicine but not conventional medicine and the clinical outcomes of this choice.

## Background

Despite national surveys suggesting that approximately 40% of the U.S. adult population use complementary medicine or alternative medicine [1-3], prior studies examining health needs and healthcare utilization in the U.S. adult population have not controlled for or considered the use of complementary medicine or alternative medicine in their calculations. It has been estimated that 16-26% of the adult population does not receive conventional care in a given year [4-9]. It has also been estimated that while most individuals who use complementary medicine or alternative medicine use it as complementary to conventional medicine (complementary medicine) [10,11], about 4% of the adult population may be using it as an alternative to conventional medicine (alternative medicine) [10,11]. Thus, up to 20-25% of the adult population not receiving conventional care in a given year might, in fact, be using alternative medicine instead.

It may be that individuals using only alternative medicine differ substantially from individuals using neither complementary medicine nor conventional care. If that is true, then prior studies investigating access to care in those not receiving conventional care may not fully reflect all relevant characteristics of this population (in that they did not distinguish alternative medicine only users). Thus, this project begins to address an Institute of Medicine observation that [12] "one of the shortcomings in the [access to care] literature is a lack of information about the experience of those adults who do not seek care, whether insured or uninsured."

This study examines whether individuals who use only alternative medicine will differ substantially in health and sociodemographic status from individuals using neither alternative medicine nor conventional care in a given year. To identify those factors that predict alternative medicine use in those not using conventional care, we employed the socio-behavioral model of healthcare utilization [13-15]. In current formulations of this framework, six sets of variables are posited to interact and influence one's use of health services: elements of the healthcare system, the external environment, predisposing factors, enabling factors, health need measures, and personal health practices. The model predicts that health needs are the most direct cause of health service use followed by enabling and predisposing factors [16]. Therefore, we hypothesize that those individuals using only alternative medicine will be less healthy and have more health needs than individuals using neither alternative medicine nor conventional care after controlling for other variables in the socio-behavioral model. We will also explore the relative contribution of predisposing (e.g., age, gender, race and ethnicity), and enabling (e.g., poverty status, marital status, health insurance coverage) factors in an individual's choice to use alternative medicine but not conventional care. These data will reveal what factors most strongly predict when an individual decides to use alternative medicine instead of conventional medicine and may help guide outreach approaches to optimize an individual's health care plans.

## Methods

To address our research questions we utilized data from the 2002 National Health Interview Survey (NHIS). The NHIS is an annual survey of the health of the U.S. civilian, non-institutionalized population conducted by the National Center for Health Statistics, Centers for Disease Control and Prevention (CDC). The 2002 survey used a multi-stage clustered sample design, and oversampled non-Hispanic black and Hispanic persons to allow for more accurate national estimates of health for these increasing minority populations.

The survey contains four main modules: Household, Family, Sample Child, and Sample Adult. The first two modules collect health and sociodemographic information on each member of all families residing within a sampled household. Within each family, additional information is collected from one randomly selected adult (the "sample adult") aged 18 years or older. For the 2002 interviewed sample, there were 36,161 households consisting of 93,386 persons in 36,831 families. The total household response rate was 89.6%. From the households interviewed, 31,044 sample adults completed interviews, resulting in an overall sample adult response rate of 74.3%.

The 2002 NHIS was approved by the National Center for Health Statistics Research Ethics Review Board on November 13, 2001. Verbal or written consent was obtained from all survey respondents (for more information on the NHIS, go to http://www.cdc.gov/nchs/nhis.htm).

### Study Population

From the pool of sample adults in the 2002 NHIS, we identified those individuals who did not report using conventional care in the previous 12 months. To be included in this group, individuals had to report not seeing any of the following medical professionals: a mental health professional such as a psychiatrist, psychologist, psychiatric nurse, or clinical social worker; a foot doctor; a nurse practitioner, physician assistant, or midwife; a doctor who specializes in women's health, such as an obstetrician or gynecologist; a medical doctor who specializes in a particular medical disease or problem; and a general doctor who treats a variety of illnesses, such as a doctor in general practice, family medicine, or internal medicine. In addition, the sample adult had to report zero trips to a hospital emergency room, not receiving care at home from a nurse or other healthcare professional, and not having surgery or other surgical procedures as an inpatient or outpatient in the past 12 months. The sociodemographics and health status of this population are presented in Table 1.

**Table 1:** Descriptive Characteristics of Sample Adults Not Using Conventional Care in the Past 12 Months^1^

Variable	All Adults	Percent Using Alternative Medicine	Chi-square **p-value**^**2**^
***External Environment***			

**Region**			<.01

**Northeast**	14.4	24.8	

**Midwest**	23.9	27.0	

**South**	37.8	21.6	

**West**	23.9	27.5	

**MSA Status**			.57

**MSA, central city**	32.5	23.9	

**MSA, non-central city**	48.0	25.5	

**Non-MSA**	19.5	24.4	

			

***Predisposing Factors***			

**Sex**			<.01

**Male**	69.5	23.3	

**Female**	30.5	28.1	

**Age**			<.001

**18-24**	18.4	21.2	

**25-44**	47.9	25.6	

**45-64**	26.6	27.1	

**65+**	7.1	19.6	

**Race/Ethnicity**			<.001

**Hispanic**	18.6	16.8	

**Non-Hispanic white**	64.2	27.6	

**Non-Hispanic black**	10.9	18.8	

**Non-Hispanic other**	6.3	30.0	

**Education**			<.001

**Less than high school**	21.9	12.6	

**High school diploma/G.E.D.**	30.5	22.3	

**Some college/AA degree**	27.7	30.7	

**Bachelor's or higher**	19.9	34.1	

**Class of Worker**			<.01

**Private sector**	58.3	24.5	

**Government**	7.5	27.9	

**Self-employed/family business**	8.8	31.6	

**Not working**	25.3	22.6	

**Born in the U.S.**			<.001

**Yes**	77.0	26.5	

**No**	23.0	19.2	

***Enabling Factors***			

**Poverty Status**			<.001

**Poor**	13.8	19.9	

**Near poor**	20.9	21.8	

**Not poor**	65.2	26.8	

**Marital Status**			.11

**Never married**	27.1	22.8	

**Married/cohabiting**	59.6	25.3	

**Divorced/separated**	10.1	27.7	

**Widowed**	3.2	22.9	

**Health Insurance Coverage**			<.001

**Uninsured**	33.5	24.7	

**Private coverage**	58.2	25.9	

**Public coverage**	8.3	17.4	

**Usual Place for Care**			.52

**Yes**	61.6	25.1	

**No**	38.4	24.3	

***Health Need Factors***			

**Reported Health Status**			.15

**Poor/fair**	5.2	21.1	

**Good/very good/excellent**	94.8	24.9	

**Health Compared to 12 Months Ago**			<.001

**Worse**	3.7	37.1	

**About the same**	83.7	22.5	

**Better**	12.6	36.5	

**Functional Limitation**			<.001

**Yes**	14.5	35.8	

**No**	85.5	22.9	

**Respondent Reported a Serious Chronic or Acute Condition?**			<.001

**Yes**	23.8	30.7	

**No**	76.2	22.6	

**Respondent Reported Back Problems**			<.001

**Yes**	17.8	37.8	

**No**	82.2	22.0	

**One or More Health Needs**^**3**^			<.001

**Yes**	39.5%	31.5	

**No**	60.5	20.0	

***Personal Health Practices***			

**Leisure-Time Physical Activity**			<.001

**Never/unable**	42.2	15.1	

**Some activity**	27.8	27.7	

**Regular activity**	30.1	35.5	

**Alcohol Drinking Status**			<.001

**Lifetime abstainer**	25.0	16.9	

**Former drinker**	11.2	27.2	

**Light/infrequent**	40.1	26.9	

**Moderate/heavy**	23.7	29.4	

**Smoking Status**			<.05

**Current smoker**	29.0	24.8	

**Former smoker**	14.5	30.1	

**Never smoked**	56.5	23.5	

***Barriers to conventional care***			

**Delayed Care Due to Cost and/or non-Cost Barriers**			<.001

**Yes**	13.2	39.1	

**No**	86.8	22.5	


^1 ^Approximately 19% (n = 5,383) of adults did not use some form of conventional care in the past 12 months.

^2 ^Chi-square analysis was used to identify statistically significant associations between the independent/control variables and the dependent variable (use of alternative medicine only versus use of neither alternative medicine nor conventional care).

^3 ^This measure is defined as any one of: poor or fair health; health is worse off than it was 12 months ago; one or more serious chronic or acute conditions; a functional limitation; and/or back problems.

### Dependent Variable

In 2002, a 10-minute supplement on complementary medicine and alternative medicine was added to the NHIS. The supplement was administered to sample adults who were asked a number of questions about the use of complementary medicine and alternative medicine therapies within the past 12 months. Alternative medicine use, the dependent variable for this study, was defined as use of any of the following in the past 12 months in those not using conventional medicine (defined above): acupuncture, Ayurveda, biofeedback, chelation therapy, chiropractic care, energy healing therapy/Reiki, folk medicine, hypnosis, massage, naturopathy, natural herbs, homeopathic treatment, diet-based therapies (specifically, Vegetarian diet, Macrobiotic diet, Atkins diet, Pritikin diet, Ornish diet and Zone diet), high dose or megavitamin therapy, yoga, tai chi, qi gong, and meditation and other relaxation techniques.

### Independent Variables

Using the socio-behavioral model of healthcare utilization [13-15], we identified several measures to be employed as independent variables in our analysis. Two measures of the external environment were included: region of residence [17], and urban/rural location or population density [18,19]. Measures of predisposing factors included age [20,21], sex [20,22], race and ethnicity [21,23], education [22,24], occupational status or class of worker [17], and immigrant status [23]. Four measures of enabling characteristics were included: poverty status [23], a source for regular healthcare [19], marital status [24], and health insurance coverage [21,22]. In the socio-behavioral model of healthcare utilization "health need" refers to an individual's level of illness, which is the most immediate cause of health service use. Therefore, we examined five measures of health needs: reported health status [25], reported changes in health status, the presence of pre-existing chronic medical conditions other than back pain [19,26], the presence of a functional limitation [27] and the presence of back pain or problems. The presence of back pain was broken out from other pre-existing medical conditions because national surveys consistently find that back pain is, by far, the most prevalent condition for which complementary medicine or alternative medicine are used [1-3,11]. In addition, a dichotomous composite measure of health need was created based on the five health need measures. More specifically, adults with one or more health needs were defined as those who had one or more of the following: poor or fair health; health is worse than it was 12 months ago; one or more serious chronic or acute conditions; a functional limitation; and/or back problems. Measures of personal health practices included the following: tobacco use [26], alcohol consumption [28,29] and level of physical activity. Finally, a dichotomous measure of barriers to conventional care was included: did the individual delay conventional care due to cost and/or non cost barriers [30].

### Statistical Analyses

Chi-square analysis was used to identify statistically significant bivariate associations (p < .05) between the independent/control variables and alternative medicine use for adults not using conventional care.

Multiple logistic regression was used to assess the relationships between the dichotomous measure of health need and use of alternative medicine in the past 12 months, after controlling for sets of external environment, predisposing factors, enabling factors and barriers to conventional care, and personal health practice controls. The models are nested in that the first regression controls for the external environment measures, the second controls for the external environment and predisposing measures and barriers to conventional care, and so on (Table 2). Variables significantly associated with the dependent variable at the .05 level in the Table 1 chi-square analysis were retained as control variables in the regressions. For each model, global Wald chi-square values were calculated, a significant value of which indicates a good-fitting model. To assess the improvement in the fit of a model with the addition of variables, we calculated the improvement chi-square. This was done by subtracting the global Wald chi-square and degrees of freedom of the previous model from the global Wald chi-square and degrees of freedom of the current model. A table of critical chi-square values was used to determine if the improvement chi-square represented a significant improvement in model fit. We also calculated adjusted odds ratios for the composite health needs measure and all other control variables included in the full model, model 4 (Table 3).

**Table 2:** Respondent Health Need and Association with Alternative Medicine Use (Versus Use of Neither Alternative Medicine nor Conventional Care

			**Model 1**^**1**^		**Model 2**^**2**^		**Model 3**^**3**^		**Model 4**^**4**^	
	
	**UOR**^**5**^	**CI**^**6**^	**AOR**^**7**^	**CI**^**6**^	**AOR**^**7**^	**CI**^**6**^	**AOR**^**7**^	**CI**^**6**^	**AOR**^**7**^	**CI**^**6**^
**One or More Health Needs**^**9**^										

Yes	1.84	1.59-2.12	1.84	1.59-2.12	1.98	1.70-2.32	1.83	1.56-2.15	1.75	1.49-2.06

No (ref)	1.00		1.00		1.00		1.00		1.00	

*Wald chi-square (d.f.; p-value)*	68.95 (1; p < .001)		91.51 (4; p < .001)		234.36 (18; p < .001)		305.55 (23; p < .001)		432.10 (30; p < .001)	

*Improvement chi-square*^8 ^*(d.f.; p-value)*	---		22.56 (3; p < .001)		142.85 (14; p < .001)		71.19 (5; p < .001)		126.55 (7; p < .001)	

^1 ^The dichotomous, composite health need measure was entered into a model controlling for the external environment (region of residence) measures significantly associated (p < .05) with the dependent variable in Table 1.

^2 ^The dichotomous, composite health need measure was entered into a model controlling for the external environment and predisposing measures (sex, age, race and ethnicity, education, class of worker, and born in the U.S.) significantly associated (p < .05) with the dependent variable in Table 1.

^3 ^The dichotomous, composite health need measure was entered into a model controlling for the external environment, predisposing measures, enabling measures (poverty status and health insurance coverage), and barriers to conventional care (delayed care due to cost and/or non-cost barriers) significantly associated (p < .05) with the dependent variable in Table 1.

^4 ^The dichotomous, composite health need measure was entered into a model controlling for the external environment, predisposing measures, enabling measures, barriers to conventional care, and personal health practice measures (leisure-time physical activity, alcohol drinking status, smoking status) significantly associated (p < .05) with the dependent variable in Table 1.

^5 ^UOR=unadjusted or crude odds ratio.

^6 ^CI= 95% confidence interval.

^7 ^AOR=adjusted odds ratio.

^8 ^Analogous to the F-change statistic in ordinary least squares regression, the improvement chi-square is a test statistic used to determine if the variables entered in each step improve the fit of the model.

^9 ^This measure is defined as any one of: poor or fair health; health is worse off than it was 12 months ago; one or more serious chronic or acute conditions (see definition in Table 1); a functional limitation (see definition in Table 1); and/or back problems.

**Table 3:** Logistic Regression Results for Socio-Behavioral Model Predicting. Alternative Medicine Use among Adults Not Using Conventional Care in the Past 12 Months

Variable	**AOR**^**1**^	**95% CI**^**2**^
**One or More Health Needs**^3^		
Yes	1.75	1.49-2.06
No	1.00	
		
***External Environment***		
**Region**		
Northeast	1.07	0.84-1.38
Midwest	1.19	0.95-1.49
South	1.00	
West	1.34	1.09-1.66
		
***Predisposing Factors***		
**Sex**		
Male	1.00	
Female	1.71	1.43-2.04
**Age**		
18-24	1.00	
25-44	1.02	0.80-1.30
45-64	1.06	0.80-1.40
65+	1.03	0.68-1.55
**Race/Ethnicity**		
Hispanic	1.01	0.78-1.31
Non-Hispanic white	1.00	
Non-Hispanic black	0.80	0.61-1.06
Non-Hispanic other	1.67	1.15-2.41
**Education**		
Less than high school	1.00	
High school diploma/G.E.D.	1.74	1.35-2.23
Some college/AA degree	2.54	1.97-3.28
Bachelor's or higher	2.95	2.20-3.95
**Class of Worker**		
Private sector	1.15	0.91-1.44
Government	0.90	0.62-1.31
Self-employed/family business	1.41	1.04-1.92
Not working	1.00	
**Born in the U.S.**		
Yes	1.25	0.96-1.63
No	1.00	
		
***Enabling Factors***		
**Poverty Status**		
Poor	1.00	
Near poor	1.06	0.78-1.44
Not poor	1.15	0.88-1.51
**Health Insurance Coverage**		
Uninsured	1.00	
Private coverage	0.83	0.68-1.02
Public coverage	0.69	0.49-0.98
		
***Personal Health Practices***		
**Leisure-Time Physical Activity**		
Never/unable	1.00	
Some activity	1.70	1.37-2.12
Regular activity	2.62	2.14-3.21
**Alcohol Drinking Status**		
Lifetime abstainer	1.00	
Former drinker	1.59	1.20-2.12
Light/infrequent	1.32	1.05-1.67
Moderate/heavy	1.58	1.22-2.06
**Smoking Status**		
Current smoker	1.02	0.84-1.23
Former smoker	1.18	0.93-1.50
Never smoked	1.00	
		
***Barriers to conventional care***		
**Delayed Care Due to Cost and/or non-Cost Barriers**		
Yes	1.92	1.55 - 2.38
No	1.00	

^1 ^AOR = adjusted odds ratio.

^2 ^CI = 95% confidence interval.

^3 ^This measure is defined as any one of: poor or fair health; health is worse off than it was 12 months ago; one or more serious chronic or acute conditions; a functional limitation; and/or back problems.

Next we determined if barriers to conventional care are present for those adults who use alternative medicine. Odds ratios were calculated using multiple logistic regression to assess the relationships between two measures of barriers to conventional care and the use of alternative medicine in the past 12 months (versus not using alternative medicine). The analysis is limited to adults with one or more health needs who reported not using conventional care in the past 12 months. All external environment, predisposing, enabling, and personal health practice measures significantly associated (p < .05) with the dependent variable via chi-square analysis (see Table 1) were entered as controls in the regression.

We conclude these analyses by presenting the reasons for using alternative medicine for treatment purposes among adults who use alternative medicine, and the types of alternative medicine therapies they used. Table 4 presents the percentage of adults reporting negative and positive reasons for using alternative medicine. Positive reasons for using alternative medicine included that alternative medicine was suggested by a conventional medical professional or that the participant thought alternative medicine would be interesting to try. Negative reasons for using alternative medicine included participants reporting that conventional medical treatments would not help or were too expensive.

**Table 4:** Reasons Persons Who Use Only Alternative Medicine for Their Healthcare Used CAM for Treatment Purposes: NHIS, 2002 (weighted)

	**Negative Reasons**^**1**^		**Positive Reasons**^**1**^	
	Conventional medical treatments wouldn't help: **% (S.E.)**^**2**^	Conventional medical treatments were too expensive: **% (S.E.)**^**2**^	Suggested by a conventional medical professional: **% (S.E.)**^**2**^	Thought it would be interesting to try: **% (S.E.)**^**2**^
**All individuals who use only alternative medicine**	21.6 (1.7)	20.4 (1.8)	13.2 (1.5)	54.1 (2.3)
**Individuals with one or more health needs who use only alternative medicine**^**3**^				
All individuals with one or more health needs	23.6 (2.4)	22.2 (2.3)	13.2 (1.9)	55.8 (2.9)
Reported a cost or non-cost barrier to conventional care	26.5 (4.1)	40.0 (5.0)	13.0 (3.2)	47.5 (5.0)
**Individuals without a health need who use only alternative medicine**	17.0 (2.8)	15.7 (2.8)	12.9 (2.7)	51.7 (3.9)

^1 ^Respondents may select more than one reason for using a alternative medicine therapy for treatment.

^2 ^S.E. =standard error

^3 ^Adults with one or more health needs were defined as those who had one or more of the following: poor or fair health; health is worse off than it was 12 months ago; one or more serious chronic or acute conditions (see definition in Table 1); a functional limitation (see definition in Table 1); and/or back problems.

Eisenberg and colleagues [10] first proposed that therapies used for both complementary medicine and alternative medicine could be dichotomized into those that typically involve a practitioner (e.g., acupuncture, chiropractic care), and those that do not (e.g., diet-based therapies). Subsequently, the National Center for Complementary Medicine and the CDC have expanded on this concept and termed these two groups of therapies as practitioner-based therapies (i,e., acupuncture, Ayurveda; biofeedback; chelation therapy; chiropractic care; energy healing therapy/Reiki; folk medicine; hypnosis; massage; and naturopathy), and self-care therapies (defined as therapies that a person can perform alone, even if some training is required, i.e., nonvitamin, nonmineral, natural products; homeopathic treatment; diet-based therapies; high dose/megavitamin therapy; yoga; tai chi; qi gong; meditation; guided imagery; progressive relaxation; and deep breathing exercises), and examined differences in the use of these two groups of therapies [31]. We therefore present prevalence estimates for practitioner-based and self-care alternative medicine therapies in Table 5. We also presents prevalence estimates for each of four different alternative medicine domains (Alternative Medical Systems, Biologically-based Therapies, Mind-body Therapies, and Manipulative and Body-based Therapies) previously examined in analyses of the 2002 and 2007 NHIS [2,3]. The therapies within each of these four domains are as follows: Alternative medical systems include acupuncture; Ayurveda; homeopathic treatment; and naturopathy. Biologically-based therapies include chelation therapy; folk medicine; nonvitamin, nonmineral, natural products; high dose/megavitamin therapy; and diet-based therapies. Mind-body therapies include biofeedback; meditation; guided imagery; progressive relaxation; deep breathing exercises; hypnosis; yoga; tai chi; and qi gong. Manipulative and body-based therapies include chiropractic care and massage. While the therapies within the four alternative medicine domains are unique to a single domain (e.g., meditation is counted only with the Mind-body Therapy domain), the therapies are also coded, as appropriate, to either Practitioner-based Therapies, or Self-care Therapies (e.g., meditation was also coded as a self-care therapy). P-values from a chi-square analysis assessing the bivariate relationships between use of each of the alternative medicine domains and having or not having a health need are also presented.

**Table 5:** Types of CAM Therapies Used by Individuals Using Only Alternative Medicine in the Past 12 Months: NHIS, 2002 (weighted)

			Alternative Medicine Domains			
			
	**Practitioner-based Therapies**^**1**^	**Self-Care Therapies**^**2**^	Alternative Medical **Systems**^**3**^	**Biologically-based Therapies**^**4**^	**Mind-body Therapies**^**5**^	**Manipulative and Body-based Therapies**^**6**^
**All individuals who use only alternative medicine**	23.6%	90.0%	7.2%	68.5%	41.0%	21.8%
						
**Individuals with one or more health needs**^**7**^						
Yes	27.0%	88.7%	7.7%	69.2%	41.2%	24.2%
No	20.1	91.3	6.7	67.9	40.8	19.2
*Chi-square p-value*^8^	<.01	.1613	.5566	.6552	.9135	<.05

^1 ^Practitioner-based therapies include acupuncture; Ayurveda; biofeedback; chelation therapy; chiropractic care; energy healing therapy/Reiki; folk medicine; hypnosis; massage; and naturopathy.

^2 ^Self-care therapies include nonvitamin, nonmineral, natural products; homeopathic treatment; diet-based therapies; high dose/megavitamin therapy; yoga; tai chi; qi gong; meditation; guided imagery; progressive relaxation; and deep breathing exercises.

^3 ^Alternative medical systems include acupuncture; Ayurveda; homeopathic treatment; and naturopathy.

^4 ^Biologically-based therapies include chelation therapy; folk medicine; nonvitamin, nonmineral, natural products; high dose/megavitamin therapy; and diet-based therapies.

^5 ^Mind-body therapies include biofeedback; meditation; guided imagery; progressive relaxation; deep breathing exercises; hypnosis; yoga; tai chi; and qi gong.

^6 ^Manipulative and body-based therapies include chiropractic care and massage.

^7 ^Adults with one or more health needs were defined as those who had one or more of the following: poor or fair health; health is worse off than it was 12 months ago; one or more serious chronic or acute conditions (see definition in Table 1); a functional limitation (see definition in Table 1); and/or back problems.

^8 ^Chi-square analyses were used to test differences in CAM use between those with and without health needs

All estimates were generated using SUDAAN software (version 9.0, Research Triangle Institute, Inc., Research Triangle Park, NC) to account for the complex sample design of the NHIS. To represent the U.S., civilian, non-institutionalized population age 18 years and over, all estimates were weighted using the NHIS sample adult record weight.

## Results

### Characteristics of Those Who Did Not Report the Use of Conventional Care

It was found that 19.3% of adults did not use conventional care within the last 12 months. This equates to roughly 38.3 million adults. Of these, 38.4% (approximately 14.7 million) had some health need, with 23.8% having a serious acute or chronic condition. Almost one-quarter (24.8%; approximately 9.5 million adults) of those not using conventional care used some form of alternative medicine, with 12.0% (approximately 4.6 million) reporting one or more health needs and using alternative medicine.

The majority of individuals not using conventional care were male, younger than 45 years old, non-Hispanic white, without a college education, married, working in the private sector, born in the U.S., were not poor, had private health insurance, had a usual place of care, had not delayed care because of cost or non-cost issues, currently drank alcohol, or were involved in some type of leisure-time physical activities (Table 1).

### Characteristics of Those Who Did Not Report the Use of Conventional Care But Did Use Some Form of Alternative Medicine

#### External Environment

Region of residence but not population density (metropolitan statistical area - MSA - status) was associated with alternative medicine use (Table 1).

#### Predisposing Factors

Sex (female), age, race and ethnicity, education, class of worker and born in the U.S. (U.S. born) were all associated with alternative medicine use (Table 1).

#### Enabling Factors

While poverty status and health insurance coverage were associated with alternative medicine use (Table 1), marital status and having a usual place of care were not.

#### Barriers to Conventional Care

Delaying care due to cost and/or non-cost barriers was associated with the use of alternative medicine.

#### Health Need Factors

For all adults not using conventional care, individuals reporting a functional limitation, a serious chronic or acute condition, back problems, or one or more health needs were more likely to use alternative medicine (Table 1). Reported changes in health status compared to 12 months ago were also associated with alternative medicine use. A greater percentage of individuals reporting improvements or declines in their health used alternative medicine than did individuals who reported their health to be about the same. However, current health status was not associated with alternative medicine use.

#### Personal Health Practices

Leisure-time physical activity, alcohol drinking status and smoking status were all associated with alternative medicine use.

### Health Need Measures and the Use of Alternative Medicine

Table 2 shows the results from a series of logistic regression models fitted for having one or more health needs in which external environment measures (Model 1), predisposing factors (Model 2), enabling factors and barriers to conventional care (Model 3), and personal health practice measures (Model 4) are added sequentially to the regression models as controlling factors.

The positive association seen between having one or more health needs and alternative medicine use in the unadjusted analysis is maintained after sequentially adjusting for the external environment, predisposing factors, enabling factors and barriers to conventional care, and personal health practice controls (models 1-4). All effects were in a similar direction, though somewhat attenuated when the personal health practice measures were added (model 4). While each model is a better fit of the data than the preceding model, the addition of predisposing factors (model 2), and personal health practices (model 4) produced the largest changes in the improvement chi-square. The addition of enabling factors and barriers to conventional care to the model (model 3) produced a moderate improvement in chi-square, along with a small attenuation of the health needs measure.

To explore the model in more detail, Table 3 presents adjusted odds ratios for the composite health needs measure and all other control variables included in the model. In general, observed associations seen in bivariate analyses (chi square) were maintained after adjusting for other independent variables with the exception of age, born in the U.S., poverty status and smoking status for which no associations were seen in the fully adjusted model. Consistent with the model building presented in Table 2, the odds ratios associated with two predisposing variables (education level and gender) were among the largest seen. Large odds ratios were also seen for personal health practices (leisure-time physical activity and alcohol drinking status) and for delaying conventional care because of either cost and/or non-cost barriers. As might be predicted from the model building presented in Table 2, individual enabling factors were either not associated with alternative medicine use (poverty status) or inversely associated with alternative medicine use (health insurance coverage).

### Barriers to Using Conventional Care in Those Who Used Only Alternative Medicine

Given the strong association between alternative medicine use and delaying conventional care, we examined the impact of cost and non-cost barriers to conventional care separately. After adjusting for external environment measures, predisposing factors, enabling factors, and personal health practices, we found that the association between alternative medicine use and non-cost barriers (AOR = 2.04; 95% CI = 1.28-3.25) was slightly stronger than that seen for cost barriers (AOR = 1.67; 95% CI = 1.22-2.30).

### Reasons People Use Alternative Medicine

Of those who used alternative medicine for treatment purposes, 59.9% had positive reasons for this use, and 33.7% had negative reasons relative to conventional care (Table 4). The most prevalent reason for alternative medicine use was that respondents "thought it would be interesting to try." For this response, there were no differences when looking at only those adults with one or more health needs.

About 20% of individuals used alternative medicine because they believed conventional treatments would not work or because conventional medical treatments were too expensive. Those with one or more health needs were significantly more likely to use alternative medicine for these two reasons than those in good health.

### Types of Alternative Medicine Therapies Used

We examined the prevalence of use of six categories of alternative medicine therapies (Table 5). For the overall sample of individuals using only alternative medicine, self-care therapies were more popular than practitioner-based therapies (90.0% vs. 23.6%).

Of the six categories, only practitioner-based therapies and manipulative and body-based therapies showed a significant difference between healthy individuals and those with one or more health needs. While 27.0% of those with one or more health needs used practitioner-based therapies, only 20.1% of healthy individuals used these therapies (p < .01). Similarly, while 24.2% of those with one or more health needs used manipulative and body-based therapies, only 19.2% of healthy individuals used these therapies (p < .05).

## Discussion

We found that 19.3% of adults (38.3 million) did not use conventional healthcare in the last 12 months despite that fact that 38.4% of these individuals had one or more health needs, with almost one-quarter having a serious chronic or acute medical condition. Instead of conventional care, 24.8% of these individuals used alternative medicine. Several striking differences were seen when comparing the characteristics of those who used alternative medicine to those who used neither alternative medicine nor conventional medicine. First, users of alternative medicine had poorer health. Second, users of alternative medicine were more likely to have more barriers to care as exemplified by their having to delay conventional care because of both cost and non-cost factors, with 1 in 5 having used alternative medicine because conventional care was too expensive. Finally, those who used only alternative medicine and those who used neither alternative medicine nor conventional medicine displayed distinctly different patterns of predisposing factors, as well as different patterns of personal health practices.

While the present data do not allow us to directly answer the question as to why the predisposing factors and personal health practices of alternative medicine users differed from non-users, they are consistent with the hypothesis that complementary medicine and alternative medicine users are more likely to have a wellness lifestyle than non-users [32]. For instance, it has been proposed that higher education, a predisposing factor strongly associated with alternative medicine use in the present study, increases an individual's exposure to various types of complementary medicine and alternative medicine therapies [11], perhaps through increased medical literacy and health information seeking activity [33]. This seems especially true for the use of modern technologies like the internet [34], which are increasingly used to access information on complementary medicine and alternative medicine [35]. It may be that positive health behaviors associated with a wellness lifestyle [32] cluster in alternative medicine users just as they do in females and those with higher education in the general population [36]. Supporting this contention, it has been found that complementary medicine and alternative medicine are associated with a number of positive health behaviors that would be part of a wellness lifestyle [32] including regular levels of exercise [37], nonuse of tobacco [37-39], nonuse or moderation in use of alcohol [37,40], healthy diet choices [40] and preventive screening [41]. While the 2002 NHIS did not specifically ask participants if they used complementary medicine or alternative medicine for wellness, the 2007 NHIS did incorporate such a question. Future planned analysis of the 2007 dataset will allow direct assessment of whether those using only alternative medicine, do so for their overall wellness, as well as to treat specific diseases or conditions.

Our analyses are consistent with other national surveys [10,11,42] showing that a relatively small proportion (1.7%-4.4%) of the population use alternative medicine but not conventional medicine. While predisposing factors (age, education, race and sex) were not predictors of relying primarily on alternative medicine in Astin's study [11], education, race and sex were associated with the use of alternative medicine among those not using conventional care in the present study. Astin cautioned that his small sample size may have missed important predictors of using only alternative medicine. While, to our knowledge, no other studies besides Astin [11] have specifically examined predictors of alternative medicine use, several studies have examined the predictors of complementary medicine and alternative medicine combined [11,37,43,44]. Consistent with the present results, these earlier studies identify education, race and sex as predictors of use.

Contrary to earlier studies on complementary medicine and alternative medicine combined [43-45], enabling factors appear to have little impact on the use of alternative medicine. Yet, cost issues seem to play some role in whether an individual uses alternative medicine versus neither complementary medicine nor conventional medicine in that even after accounting for insurance coverage and poverty status, those who delayed conventional care because of cost were more likely to use alternative medicine. It is, therefore, not surprising that individuals who used only alternative medicine predominately used lower cost self-care therapies such as dietary supplements and mind-body therapies. However, when faced with one or more health needs, individuals who used only alternative medicine were more likely to use practitioner-based therapies, especially manipulative and body based therapies.

While cost and other barriers to conventional care may be motivators of alternative medicine use, there also is an indication that some users of only alternative medicine do not find conventional medicine helpful (Table 5). These data are consistent with observations suggesting that individuals who use only alternative medicine distrust the conventional care system and are generally dissatisfied with conventional care [11,46,47]. In a similar vein, skepticism toward medical care is strongly associated with reduced use of conventional healthcare, even after controlling for predisposing, enabling and need factors [48]. For these individuals, the value of conventional care for their health needs may not be appreciated.

Some users of only alternative medicine used alternative medicine because they felt conventional care was too expensive. It is possible this group would use conventional care if they could. Future research might assess whether this population is aware of public health insurance options and other failsafe measures to pay for conventional care. Finally, a substantial proportion of respondents who used only alternative medicine did so because they thought it would be interesting to try. Some of these individuals may be encountering non-cost barriers to conventional care, while, as mentioned earlier, others appear to be using alternative medicine as part of a healthy lifestyle choice [11,32].

Our study has several limitations. First, the cross sectional nature of the study does not allow us to assess clinical outcomes in our two identified populations, those who use neither alternative medicine nor conventional healthcare and those who use only alternative medicine. Thus we cannot comment on the potential safety or efficacy of using only alternative medicine. Given that substantial numbers of both groups have one or more health needs, future prospective studies of these populations should investigate a number of possible outcomes such as avoidable hospitalization or premature death. Second, our measures were based on self-reported data that were not independently verified. Third, many other factors that may enable or impede healthcare utilization are not measured in this report but need to be considered. These factors include health beliefs, cultural practices, language barriers, social networks and contacts, and the availability of care in the community [15]. Fourth, we limited our population for analysis to those individuals who did not report seeing a conventional provider in the preceding 12 months. There is always the potential for recall error in these types of questions. Finally, because our primary focus was to identify factors associated with the use, versus nonuse, of alternative medicine, a dichotomous dependent variable was utilized. By doing so, information on the number and type of alternative medicine therapies used and frequency of their use was lost. It may be that substantial differences exist between heavy and light users of alternative medicine or between the various, heterogeneous alternative medicine modalities.

## Conclusion

We found that a quarter of individuals who forgo conventional care in a given year instead turn towards alternative medicine. Overall, our study suggests that while the potential determinants of using alternative medicine but not conventional care are multifactorial, healthcare needs followed by predisposing factors are prime drivers of use. Since the 2007 NHIS also contained an extensive set of supplemental questions asking about alternative medicine use, we plan longitudinal assessments comparing the 2002 and 2007 NHIS to identify any cohort or secular trends in the associations that are not evident in the cross-sectional analysis. Future research is also needed to examine the decision process behind an individual's choice to use alternative medicine but not conventional medicine and the clinical outcomes of this choice.

## Competing interests

All authors were employees of the federal government during the planning, analysis and writing of this manuscript, and performed their work as part of their official duties. No outside financial support was provided. All authors declare they have no competing interests.

## Authors' contributions

RLN conceived and designed the study, interpreted data, and drafted substantial portions of the manuscript. JMD participated in survey design, statistical analysis and data interpretation, and drafted substantial portions of the manuscript. BJS participated in survey design, statistical analysis and data interpretation, and drafted substantial portions of the manuscript.

All authors read and approved the final manuscript.

## Pre-publication history

The pre-publication history for this paper can be accessed here:

http://www.biomedcentral.com/1472-6963/10/220/prepub
